# Does cleanliness influence moral judgments? Response effort moderates the effect of cleanliness priming on moral judgments

**DOI:** 10.3389/fpsyg.2014.01276

**Published:** 2014-11-06

**Authors:** Jason L. Huang

**Affiliations:** Department of Psychology, Wayne State UniversityDetroit, MI, USA

**Keywords:** priming, moral judgment, response effort, replication, cleanliness

## Abstract

Whether cleanliness influences moral judgments has recently become a topic of debate in the psychological literature. After the initial report that activating the notion of physical purity can result in less severe moral judgments (Schnall et al., 2008a), a direct replication (Johnson et al., 2014a) with much larger sample sizes failed to yield similar findings. The current paper examines the possibility that only non-conscious activation of the cleanliness concept, as achieved in participants with low response effort on priming materials, can produce the expected effect. An online replication (Study 1, *N* = 214) provided evidence that, when participants exerted low (yet still acceptable) levels of response effort to the experimental material, cleanliness priming led to more lenient moral judgments than neutral priming. An online experiment (Study 2, *N* = 440; replicated in Study 2a, *N* = 436) manipulating participants’ effort on the priming task (low vs. high) supported the hypothesized mechanism. Specifically, respondents in the low response effort group were instructed to complete the priming task as quickly as possible without too much attention, and the cleanliness priming resulted in less extreme moral judgments than the neutral condition as expected. In contrast, respondents in the high response effort group were instructed to perform to the best of their ability on the priming task, with a non-significant difference on moral ratings between cleanliness and neutral conditions. In addition to helping resolve the controversy regarding the cleanliness hypothesis, the current paper calls into attention the role of response effort in the execution and replication of priming studies.

## INTRODUCTION

“Does cleanliness impact judgments of morality?” With this question, Johnson et al. (2014a; hereafter JCD) began their report of an unsuccessful replication of Schnall et al. (2008a; hereafter SBH). As the first published empirical work addressing this question, SBH proposed that feeling clean would lead individuals to make more lenient moral judgments (hereafter referred to as *the cleanliness hypothesis*). Their hypothesis extends earlier research (Schnall et al., 2008b) that documented a linkage between the experience of disgust—which evolved to help one avoid physical impurity (see Rozin et al., 1999)—and subsequent harsher moral judgments (i.e., avoidance of moral impurity). SBH reasoned that the experience of cleanliness would achieve the opposite effect of disgust on moral judgments. Using a sentence unscrambling task (Experiment 1, *N* = 40) and a hand-washing manipulation (Experiment 2, *N* = 43), SBH showed that, in general, participants in the cleanliness condition responded less severely to six moral vignettes than those in the neutral condition. In sharp contrast, JCD conducted a direct replication with much larger sample sizes (*N*_Experiment1_ = 208; *N*_Experiment2_ = 126) and failed to find any support for the cleanliness hypothesis. The unsuccessful replication (also see Johnson et al., 2014b; Lee, unpublished manuscript) has led to heated debates and discussions in the broad scientific community (e.g., Bohannon, 2014; Schnall, 2014; Yarkoni, 2014).

Unsuccessful replications are not unique to the effect of cleanliness priming on moral judgments. For example, the association between moral impurity and the subsequent desire for cleanliness, termed as the Macbeth Effect (Zhong and Liljenquist, 2006), has received several failed replications (e.g., Fayard et al., 2009; Earp et al., 2014). Indeed, behavioral priming effects in general have been the subject of increased scrutiny (see Cesario, 2014), and researchers have suggested different causes for failed replication, such as measurement and sampling errors (Stanley and Spence, 2014), variation in subject populations (Cesario, 2014), discrepancy in operationalizations (Stroebe and Strack, 2014), and unidentified moderators (Dijksterhuis, 2014). One can certainly conjecture that a failed replication study, such as JCD, may have been influenced by any of the factors above, but only a systematic investigation can advance understanding of the focal phenomenon. The current paper is an attempt to act upon failed replications to identify a moderator that explains discrepant findings regarding the cleanliness hypothesis.

Based on the recognition that priming effects occur non-consciously (Tulving and Schacter, 1990; Wheeler et al., 2007), I propose that the cleanliness priming will be effective among participants exerting low but acceptable levels of response effort and ineffective among those exerting high levels of response effort. Building on two pilot studies with prior failed replication data, I conducted two experiments to test the hypothesis that response effort moderates cleanliness priming’s effect on moral judgments.

This paper contributes to the psychological literature in three significant ways. First, the present studies help resolve the debate surrounding the cleanliness hypothesis (see Johnson et al., 2014b; Schnall, 2014) and as a result shed light on *when* cleanliness impacts judgments of morality. Second, identifying response effort as a moderator of cleanliness priming’s effect opens the door for studying response effort as a potential moderator for other failed priming replications. Finally, the current paper serves as a case that reminds scholars of the value of replication studies. When investigated properly, replication studies afford the opportunity and impetus to identify unknown moderating variables.

### RESPONSE EFFORT AND PRIMING EFFECT

Despite a lack of well-developed theories of priming (Cesario, 2014), scholars have generally recognized that priming effects occur automatically outside of consciousness, while conscious processes may in certain situations “override the effects of primes on behavior” (p. 257; Wheeler et al., 2007). The degree to which study participants process priming information consciously may depend on their *response effort*, namely the degree to which participants devote cognitive resources to the study material. On the extreme low end of the continuum lies *insufficient effort responding* (IER), which occurs when “the respondent answers a survey measure with low or little motivation to comply with survey instructions, correctly interpret item content, and provide accurate responses.” (Huang et al., 2012, p. 100). Within the majority of compliant respondents who provide valid data, response effort can still differ, ranging from satisficing to maximizing (see Simon, 1955, 1956). Even with the same standardized experimental instruction such as a priming manipulation, some respondents may exert greater response effort and thus engage in more conscious processing of the priming material than others.

Of direct relevance to the current investigation is the design of SBH’s Experiment 1. Noting that the cleanliness concept may be primed in a subtle fashion, Schnall et al. (2008a) asked participants to work on a sentence unscrambling task involving either cleanliness or neutral words before rating six moral vignettes. The cleanliness group gave lower moral ratings than the neutral group, *d* = -0.60, *p* = 0.06, *N* = 40, with significant difference on one of the six scenarios. In contrast, JCD argued the activation of the cleanliness concept may accentuate one’s own feelings of virtue, thus resulting in a contrast effect that brings harsher judgments on others’ morality. Their replication of Schnall et al.’s (2008a) Experiment 1 showed no difference across the two priming conditions, *d* = -0.01, *p* = 0.95, *N* = 208.

Despite being a direct replication of SBH, JCD differed from SBH on at least two subtle aspects that might have resulted in a slightly higher level of response effort. First, whereas undergraduate students from University of Plymouth in England “participated as part of a course requirement” in SBH (p. 1219), undergraduates from Michigan State University in the United States participated in exchange of “partial fulfillment of course requirements or extra credit” in JCD (p. 210). It is plausible that students who participated for extra credit in JCD may have been more motivated and attentive than those who were required to participate, leading to a higher level of response effort in JCD than in SBH. Second, JCD included quality assurance items near the end of their study to exclude participants “admitting to fabricating their answers” (p. 210); such features were not reported in SBH. It is possible that researchers’ reputation for screening for IER resulted in a more effortful sample in JCD.

Entertaining the conjecture above that JCD did not find support for the cleanliness hypothesis due to a higher level of response effort in their study, I explored two replication datasets to identify subsamples with low response effort and assessed the cleanliness hypothesis in these extreme groups. First, I explored JCD’s Experiment 1 data to identify a low response effort subsample (*n* = 57) based on percentage of correct responses on sentence unscrambling task and found tentative support for the cleanliness hypothesis [*d*_composite_ = -0.41, 95% CI (-0.94, 0.13), see Supplementary Material, Pilot Study 1]. I also explored Lee’s (unpublished manuscript) online replication data based on Mechanical Turk (Mturk) and showed similar tentative support for the cleanliness hypothesis in a low response effort subsample based on short survey duration [*d*_composite_ = -0.69, 95% CI (-1.45, 0.08), *n* = 28 out of 90; see Supplementary Material, Pilot Study 2)]. It should be noted that these pilot results, albeit promising, were highly exploratory, and thus should not be interpreted as confirmatory evidence. Instead, these tentative results point to the need for a comprehensive examination of the role of response effort.

Based on the pilot results, one may derive the expectation that the cleanliness hypothesis will receive support among low response effort individuals. Given the overall null findings in JCD and Lee (unpublished manuscript), one can also speculate that high response effort may highlight the cleanliness concept in individuals’ consciousness and thus amplify the contrast effect per JCD, which counteracts SBH’s cleanliness effect. Below, I report two experiments that examine the role of response effort in the study of the cleanliness hypothesis.

Specifically, Study 1 measured participants’ naturally occurring response effort and tested the expectation that the cleanliness priming will lead to less severe moral judgments than the neutral priming among low response effort participants. Building on findings from Study 1, Study 2 manipulated respondents’ effort directly to test the hypothesis that response effort will moderate the cleanliness priming’s effect on moral judgments.

### CURRENT REPLICATION CONSIDERATIONS

Using materials obtained from JCD, the current studies replicated SBH’s Experiment 1. Two design characteristics differed from SBH. First, the current studies were conducted online with paid participants from Mturk. With solid study design, Mturk allows for speedy collection of high-quality data (Buhrmester et al., 2011). To increase the proportion of participants that satisfice rather than maximize their response effort, the payment for the studies was set deliberately low (around $2 per hour). Second, the anonymous online environment gave rise to rapid IER (Huang et al., 2012), which would otherwise be noted by researchers in laboratory sessions. Rapid IER is a particular concern due to the low pay rate of the study. Thus, a planned exclusion scheme was in place to screen individuals with rapid IER. For all the studies below, all measures, conditions, data exclusions, and sample size determinations have been reported.

## STUDY 1

### MATERIAL AND METHODS

This research was approved by the Institutional Review Board at Wayne State University. Participants in this research provided their consent online by checking a response option indicating their agreement to participate.

#### Participants

268 English-speaking adults residing in the United States completed the current survey as a task on Mturk in exchange for $0.50 compensation. Of these, 214 participants (*n*_cleanliness_ = 111, *n*_neutral_ = 103; mean age = 35 years; 55% female; 72% white) were retained in the analysis after planned exclusion (see below) due to guessing of the study hypothesis or IER on the moral judgment items. The sample size consideration was based on JCD’s power analysis (i.e., ≥208 individuals after planned exclusion).

#### Procedure

The primary replication survey followed the procedure of SBH’s Experiment 1 obtained from JCD. Upon giving an online consent to participate, each respondent had an hour to complete the Mturk study. Participants performed a 40-item sentence unscrambling task, evaluated six moral judgment vignettes, and rated their emotional states. Similar to the procedure in JCD, participants also responded to an exploratory measure on private body consciousness (PBC; Miller et al., 1981). After the primary survey, respondents also filled out the same quality screening items used in JCD, as well as an additional scale for IER (see Measures section below). Finally, they filled out several demographic items. The average survey completion time was approximately 15 min, reflecting an average hourly pay rate of 2$.

Participants were randomly assigned into the neutral vs. cleanliness conditions, which differed only on the sentence unscrambling tasks they completed (neutral words vs. cleanliness-related words). On the sentence unscrambling task, participants in both conditions were instructed, in capital letters, to “work quickly” and to “underline words according to your first impression.”

Three exceptions differentiated the current study from SBH and JCD. First, the study was conducted online instead of using paper-and-pencil in individual sessions in a laboratory. Second, in the sentence unscrambling task, participants checked boxes underneath possible words as opposed to underlining them on paper. Finally, the survey was implemented on multiple webpages, which prevented respondents from going back to a previous page.

#### Measures

The primary replication measures were identical to SBH and JCD. The six moral vignettes included: (a) “dog,” eating one’s dog that died of an accident; (b) “trolley,” switching the direction of a runaway trolley to kill a workman instead five others; (c) “wallet,” keeping the money from a lost wallet found in the street; (d) “plane,” killing and eating an injured survivor of a plane crash to avoid starvation; (e) “résumé,” falsifying one’s resume to find employment; and (f) “kitten,” using one’s kitten for sexual arousal. After reading each vignette, respondents rated their *moral judgments* on a 10-point scale ranged from 0 (perfectly ok) to 9 (extremely wrong). A moral judgment composite was created by averaging the ratings across these six vignettes, Cronbach’s *α* = 0.59. The internal consistency reliability for the composite measure was low but comparable to the values calculated from SBH’s and JCD’s *Study 1* data (Cronbach’s *α* = 0.66 and 0.48, respectively).

SBH included nine *emotional states* to rule out any effect of the manipulation on emotions. The same items (*relaxed, angry, happy, sad, afraid, depressed, disgusted, upset,* and *confused*) were used in this study. The participants clicked on visual analog scales to indicate their emotional states, which were automatically scored from 0 to 100.

JCD included a private body conscientiousness scale as a potential moderator of the cleanliness priming’s effect. Although not part of the current study, the same 15 items were included to closely replicate JCD’s procedure.

Near the end of the survey, the survey *quality screening items* included the same open-ended questions (e.g., “could you please describe what you understood to be the purpose of this study?”) and two quality control items (“I responded to this survey honestly” and “All of my answers are made up”) as in JCD.

In addition, at the end of the survey, five IER items (e.g., “Eat cement occasionally,” “Can run 2 miles in 2 min”; see Huang et al., 2014) were scattered in 20 personality filler items with a five-point Likert response (1 = Very Inaccurate; 5 = Very Accurate).

*Response effort* was operationalized with survey duration (accurate to the second). Specifically, low response effort was defined as falling below the median on survey duration.

#### Planned exclusion

Similar to JCD, I excluded respondents who guessed cleanliness might be part of the study hypothesis (i.e., mentioning cleanliness-related words in response to open-ended quality control items; *n* = 2). Furthermore, to guard against IER behavior on the focal measures, I included the page time measure (Huang et al., 2012) for planned exclusion. Specifically, each vignette was presented on a webpage with submission time automatically recorded, and respondents were excluded (*n* = 54; 20% of the total sample) if they responded faster than 675 words per minute (wpm) on two or more moral vignettes. 675 wpm resembles the reading speed of an average college professor, while the average adult’s reading speed is around 200–340 wpm (Kershner, 1964; Nelson, 2012). Because the respondents also needed to spend time reading the response scale, selecting the appropriate options, and submitting the responses, the current estimates represented conservative estimates of their actual reading speed. It is also worth noting that the number of times a respondent exceeded the 675 wpm limit was strongly associated with his/her score on the IER scale (*α* = 0.79) before the exclusion, *r* = 0.55, *p* < 0.001.

One consideration led to the current decision to not exclude participants based on quality control items embedded at the end of the survey. Whereas the page time approach described above assessed IER on the moral judgments, the quality control items reflected IER near the end of the survey. As participants progressed toward the end of the survey, more IER may emerge due to lack of interest, fatigue, or distraction (see Clark et al., 2003), which may not influence the study results after screening on the page time. Rather than potentially overscreening respondents using the IER scale and JCD’s two quality control items, I applied these exclusion criteria in supplementary analyses (see Supplementary Material), which did not alter the current results.

### RESULTS

I conducted an independent samples *t*-test to examine the overall effect of cleanliness priming on the severity of composite moral judgments. Although in the hypothesized direction, the effect was non-significant, *t*(212) = -1.22, *p* = 0.23, *d* = -0.17. Following SBH and JCD, I performed *t* test on each individual vignette. At the individual item level, *d* between conditions ranged from 0.10 to -0.24 (see **Table 1**). Taken at face value, the results would represent another failure to replicate the original study. In addition, similar to SBH, independent samples *t*-tests revealed no difference between priming conditions on any of the emotions, *t*s ranged from -1.49 to 0.73, *p* ranged from 0.14 to 0.99.

**Table 1:** Mean ratings for moral vignettes in study 1.

Condition	Composite rating	Individual vignette rating					
		Dog	Trolley	Wallet	Plane	Résumé	Kitten
**Full sample, *n*_cleanliness_ = 111, *n*_neutral_ = 103**	
Cleanliness	6.00 (1.54)	6.52 (2.74)	2.73 (2.30)	6.63 (2.65)	7.14 (2.54)	5.86 (2.76)	7.13 (2.55)
Neutral	6.24 (1.39)	6.76 (2.78)	3.27 (2.63)	6.36 (2.61)	7.17 (2.45)	6.49 (2.43)	7.43 (2.26)
*t*	-1.22	-0.62	-1.60	0.76	-0.09	-1.77^†^	-0.91
*p*	0.23	0.54	0.11	0.45	0.93	0.08	0.36
*D*	-0.17	-0.09	-0.22	0.10	-0.01	-0.24	-0.12
*d*_LL_	-0.43	-0.35	-0.49	-0.17	-0.28	-0.51	-0.39
*d*_UL_	0.10	0.18	0.05	0.37	0.26	0.03	0.14
**Identified low response effort subsample, *n*_cleanliness_ = 50, *n*_neutral_ = 57**	
Cleanliness	5.54 (1.58)	6.14 (2.76)	2.40 (1.62)	5.78 (2.89)	6.56 (2.62)	5.48 (2.94)	6.88 (2.69)
Neutral	6.15 (1.40)	6.65 (2.75)	3.16 (2.66)	6.37 (2.67)	6.81 (2.48)	6.49 (2.51)	7.40 (2.42)
*t*	-2.11*	-0.95	-1.75^†^	-1.10	-0.50	-1.92^†^	-1.06
*p*	0.04	0.34	0.07	0.28	0.62	0.06	0.29
*D*	-0.41	-0.18	-0.35	-0.21	-0.10	-0.37	-0.21
*d*_LL_	-0.79	-0.57	-0.73	-0.59	-0.48	-0.75	-0.59
*d*_UL_	-0.02	0.20	0.03	0.17	0.28	0.01	0.18

*p< 0.05;_†_*p*< 0.10.Standard deviations are presented in parentheses.

*D_LL_ and *d*_UL_: lower and upper limits of the 95% CI for *d*, computed using syntax provided by Wuensch (2012).*

Next, I created the low response effort subsample (*n* = 107) based on the median of survey duration. Although artificially dichotomizing a continuous variable usually results in loss of information, the current use of the median was justifiable because it was intended to identify an extreme group (see DeCoster et al., 2009) that, based on the two pilot studies, represented approximately a third of the entire sample. Consistent with the expectation, the cleanliness condition had lower composite judgment scores than the neutral condition in this subsample, *t*(105) = -2.11, *p* = 0.04, *d* = -0.41. Standardized difference on the individual vignettes ranged from -0.10 to -0.37 (see **Table 1**).

Although the cleanliness hypothesis was supported in the low response effort subsample, it is worth noting that response effort, as measured with survey duration, did not moderate the effect of the cleanliness priming. Specifically, a moderated regression analysis (with grand-mean centered duration) revealed a non-significant cleanliness × duration interaction, *B* = 0, β = 0.14, Δ*R*^2^ = 0.01, *p* = 0.21. Similarly, a 2 (neutral vs. cleanliness) × 2 (low vs. high dichotomized response effort) factorial ANOVA yielded a non-significant interaction effect, *F_Cleanliness_*_×_ *_Effort_* = 2.43, $\eta_{p}^{2}$ = 0.01, MSE = 2.08, *p* = 0.12.

### DISCUSSION

Study 1 supported the expectation that the cleanliness priming will result in less severe moral judgments than the neutral condition among low response effort participants. Combined with the pattern of results from the two pilot studies, Study 1’s findings suggest that the cleanliness hypothesis may hold true in individuals exerting low (but acceptable) survey response effort. However, the correlational nature of Study 1 does not allow causal inference. Moreover, response effort, as measured by survey duration, did not significantly interact with the cleanliness priming to influence moral judgments. One might argue that the design of Study 1 precluded an actual high response effort subsample that would allow for a direct test of response effort’s moderating role. An experimental study manipulating response effort can enable a stronger inference, with the expectation that the cleanliness priming effect will be stronger for participants instructed to input low response effort on the sentence unscrambling task, as compared to those instructed to maximize effort.

The instruction for the sentence unscrambling task contained a sentence that read “WORK QUICKLY,” which may give rise to different interpretations. Participants with low response effort may view this instruction literally and even see it as an opportunity to complete the study as quickly as possible. In contrast, those with high response effort may still emphasize quality of response over speed, and thus process the information too consciously. Thus, this focal sentence was modified to induce two levels of response effort on the priming task in Study 2.

## STUDY 2

### MATERIALS AND METHOD

This research was approved by the Institutional Review Board at Wayne State University. Similar to Study 1, participants in this research provided their consent online by checking a response option indicating their agreement to participate.

#### Participants

535 English-speaking adults residing in the United States (after removing 23 responses from repeat participants based on worker ID and IP address) recruited from Mturk completed this online survey in exchange for $0.50. Of these, 440 (*n*_cleanliness_ = 211, *n*_neutral_ = 229; mean age = 37 years; 63% female; 75% white) were retained in the analysis after planned exclusion based on the same criteria as in Study 1. Sample size consideration was similar to the one in Study 1 (i.e., ≥ 208 individuals in each response effort condition after planned exclusion).

#### Procedure

The study was identical to Study 1 with one exception. Participants were randomly assigned into one of two response effort instructional sets for the sentence unscrambling task. Those in the low response effort condition (*n* = 218) read “Work as quickly as you can. Select words according to your first impression and do not worry about getting everything right.” In contrast, those in the high response effort condition (*n* = 222) read “Work quickly. Select words according to your first impression but perform to the best of your ability. The data will be screened for excessive errors.”

#### Measures

The survey measures in the current study were identical to those in Study 1. The moral judgment composite had an internal consistency reliability of 0.65.

#### Planned exclusion

Respondents were screened for the same two criteria as in Study 1. Fifteen respondents were excluded for having guessed the purpose of the study, while another 80 (15%) were excluded for excessively fast moral judgment. Similar to Study 1, supplementary analysis based on a set of more stringent exclusion criteria revealed the same pattern of results (see Supplementary Material).

### RESULTS

As an initial step of the analysis, an independent samples *t*-test on the composite moral ratings across the cleanliness and neutral conditions revealed non-significant difference, *t*(438) = -0.42, *p* = 0.68, *d* = -0.04. Similar to Study 1, the overall result from Study 2 would suggest that the cleanliness priming did not have the hypothesized impact on moral judgment. Further investigation of standardized difference across priming conditions on individual vignettes ranged from -0.21 to 0.11 (see **Table 2**), with a significant difference on the “*dog*” vignette, *t*(438) = -2.26, *p* = 0.02, *d* = -0.21. That is, participants in the cleanliness condition rated that particular vignette as less morally offensive than those in the neutral condition.

**Table 2:** Mean ratings for moral vignettes in study 2.

Condition	Composite rating	Individual vignette rating					
		Dog	Trolley	Wallet	Plane	Résumé	Kitten
**Full sample, *n*_cleanliness_ = 211, *n*_neutral_ = 229**	
Cleanliness	6.20 (1.54)	6.50 (2.90)	3.00 (2.37)	6.89 (2.54)	7.27 (2.35)	6.10 (2.73)	7.45 (2.42)
Neutral	6.26 (1.59)	7.07 (2.67)	3.10 (2.71)	6.59 (2.80)	7.13 (2.46)	6.17 (2.75)	7.54 (2.26)
*t*	-0.42	-2.26*	-0.47	1.26	0.68	-0.19	-0.43
*p*	0.68	0.02	0.64	0.21	0.50	0.85	0.69
*D*	-0.04	-0.21	-0.04	0.11	0.06	-0.02	-0.04
*d*_LL_	-0.23	-0.39	-0.23	-0.07	-0.12	-0.21	-0.23
*d*_UL_	0.15	-0.02	0.14	0.30	0.25	0.16	0.15
**Instructed low response effort condition, *n*_cleanliness_ = 99, *n*_neutral_ = 119**	
Cleanliness	6.08 (1.48)	6.69 (2.74)	2.77 (2.12)	6.57 (2.62)	7.08 (2.29)	6.00 (2.61)	7.38 (2.55)
Neutral	6.51 (1.50)	7.65 (2.18)	3.12 (2.81)	6.92 (2.73)	7.15 (2.61)	6.45 (2.72)	7.76 (2.04)
*t*	-2.10*	-2.82**	-1.05	-0.96	-0.21	-1.23	-1.20
*p*	0.04	0.005	0.30	0.34	0.83	0.22	0.23
*D*	-0.29	-0.38	-0.14	-0.13	-0.03	-0.17	-0.16
*d*_LL_	-0.55	-0.65	-0.41	-0.40	-0.30	-0.43	-0.43
*d*_UL_	-0.02	-0.11	0.12	0.14	0.24	0.10	0.10
**Instructed high response effort condition,** ***n***_**cleanliness**_ = **112,** ***n***_**neutral**_ = **110**	
Cleanliness	6.31 (1.60)	6.33 (3.03)	3.20 (2.57)	7.18 (2.45)	7.45 (2.40)	6.20 (2.83)	7.50 (2.32)
Neutral	6.00 (1.65)	6.46 (3.00)	3.09 (2.61)	6.24 (2.85)	7.10 (2.30)	5.86 (2.77)	7.30 (2.47)
*t*	1.41	-0.42	0.28	2.77**	1.15	0.96	0.62
*p*	0.16	0.67	0.78	0.01	0.25	0.34	0.53
*D*	0.19	-0.04	0.04	0.35	0.15	0.12	0.08
*d*_LL_	-0.07	-0.31	-0.22	0.09	-0.12	-0.14	-0.18
*d*_UL_	0.45	0.22	0.30	0.62	0.41	0.38	0.35

**p < 0.01; *p < 0.05. Standard deviations are presented in parentheses.

*d_LL_ and d_UL_: lower and upper limits of the 95% CI for d, computed using syntax provided by Wuensch (2012).*

Independent samples *t*-tests were also performed to assess difference across priming conditions on emotions. Unexpectedly, the cleanliness condition reported a higher level of happy emotion than the neutral condition, *t*(438) = 2.37, *p* = 0.02, *d* = 0.23, with no difference across conditions on the other emotions, *t*s ranged from –1.94 to 1.81, *p* ranged from 0.05 to 0.19.

**FIGURE 1:**
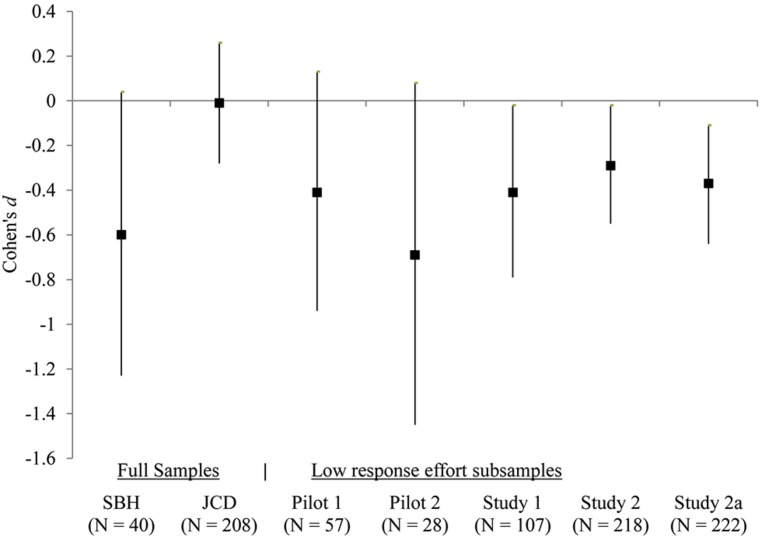
**Effect sizes of the cleanliness priming across studies.** Markers and vertical lines indicate Cohen’s *d*s and their 95% confidence intervals.

I conducted a 2 (neutral vs. cleanliness) × 2 (low vs. high response effort) factorial ANOVA to assess the hypothesis. The ANOVA revealed two non-significant main effects (*F*_priming_ = 0.16, $\eta_{p}^{2}$ = 0; *F*_effort_ = 0.87, $\eta_{p}^{2}$ = 0; MSE = 2.43) and a significant interaction (*F* = 6.05, $\eta_{p}^{2}$ = 0.01). Follow-up simple effects supported the hypothesis: The cleanliness condition had a lower mean composite rating than the neutral condition, *d* = -0.29, *p* = 0.04 in the low response effort condition, whereas the trend was reversed but non-significant in high response effort condition, *d* = 0.19, *p* = 0.16 (see **Table 2**). **Figure 1** presents the effect size estimates in the low response effort subsamples (Pilot studies 1–2; Studies 1–2), together with estimates from SBH and JCD.

A further examination of the cleanliness hypothesis on individual vignettes in the low response effort condition revealed a pattern consistent with the expectation, with *d*s ranging from –0.03 to -0.38 (see **Table 2**). In particular, the cleanliness priming resulted in a significantly less extreme rating on the “*dog*” scenario than the neutral condition, *d* = -0.38, *p* = 0.005. In contrast, in the high response effort condition, the cleanliness priming tended to have more severe ratings than the neutral condition on individual vignettes, *d*s ranged from -0.04 to 0.35, with the difference reaching statistical significance for the “*wallet*” vignette. Recall that JCD argued that cleanliness may lead to a contrast effect and hence harsher judgments on others. Although not a focus of the current study, results from this condition provided tentative support for the contrast effect.

An additional analysis was conducted to examine whether the two response effort conditions differ on survey duration, as one might expect participants in the high response effort condition to spend longer time than those in the low response effort condition. An independent *t*-test, however, revealed non-significant difference between the condition, *t*(438) = 0.54, *d* = 0.05, *p* = 0.59.

### DISCUSSION

Unlike Study 1 that measured naturally occurring response effort, Study 2 manipulated response effort on the sentence unscrambling task. Support for the cleanliness hypothesis was found among participants instructed to work through the sentence unscrambling task as quickly as possible. In contrast, when participants were instructed to work on the sentence unscrambling task attentively, no support was found for the hypothesized effect of the cleanliness priming.

Several decisions in the analyses above presented opportunities for researcher degrees-of-freedom that may result in false positive findings (Simmons et al., 2011). As requested by a reviewer, I conducted one confirmatory direct replication of Study 2 to address such a potential concern. Results reported in the Supplementary Material (Study 2a, *N* = 436) again supported the moderating role of response effort.

## GENERAL DISCUSSION

Results from Study 1 and Study 2 corroborate each other to indicate that response effort serves as a boundary condition for the effect of SBH’s cleanliness priming on moral judgment. When participants expended low levels of attention and effort on the study material, whether due to naturally occurring individual differences (Study 1) or due to experimental instruction (Study 2), SBH’s cleanliness priming resulted in less severe moral judgments than the neutral condition.

These results provide additional insight on the recent debate surrounding the unsuccessful replications of SBH. Just as Johnson et al. (2014b) acknowledged that “No two studies are perfectly identical,” the current findings indicate that the failed replications reported in JCD and Lee (unpublished manuscript) could be due to higher response effort in these studies. The current estimates also echo the effect size estimate (*d* = -0.46, *N* = 60) from an unpublished conceptual replication of SBH’s Experiment 1, where different words were used in the sentence unscrambling task (Besman et al., unpublished manuscript). Together, the cumulative evidence suggests that the effect of cleanliness priming in the form of sentence unscrambling task lies somewhere between the estimates from SBH and JCD (see **Figure 1**).

Beyond the immediate contribution to resolving the controversy surrounding the cleanliness hypothesis, the current paper suggests that response effort may play an important role in priming studies in general. Researchers may want to investigate response effort as a moderator in failed replications of other priming studies. Specifically, researchers should pay close attention to subtle differences in study features such as experimental instructions, survey administration, participant rapport building, and research incentives that may lead to different levels of response effort across studies and in turn discrepant findings for priming effects. Meanwhile, the moderating role of response effort also calls into question the value of meta-analyzing a particular effect of interest. Although meta-analysis can estimate an overall effect as well as help identify potential moderators, it may be challenging to correctly identify and code response effort level at the study level. As a result, if a meta-analysis is conducted, the overall estimate will be influenced by the response level of each primary study included.

From a methodological viewpoint, the non-significant effect of the response effort manipulation on survey duration in Study 2 (and Study 2a, see Supplementary Material), coupled with Study 1’s finding that survey duration did not moderate the effect of the cleanliness priming, appears to suggest that survey duration serves only as a coarse indicator of response effort. Indeed, although short survey duration may reflect satisficing response behavior (after removing rapid IER), long survey duration may capture both effortful and distracted responding. Thus, researchers may want to devise a granular measure of response effort that is sensitive to slight variation in effort and attention.

Finally, the current investigation makes a case for replication in advancing psychological science. As the field of psychology has only recently started to embrace replication (e.g., Pashler and Wagenmakers, 2012), uncertainty and tensions can sometimes emerge when replication results are inconsistent with original research reports (Bohannon, 2014). The current research serves as an example that replications can point researchers to potential moderating mechanisms that will otherwise be ignored.

## Conflict of Interest Statement

The author declares that the research was conducted in the absence of any commercial or financial relationships that could be construed as a potential conflict of interest.
