# Supplementary material for: Does cleanliness influence moral judgments? Response effort moderates the effect of cleanliness priming on moral judgments
Source: Front Psychol. 2014 Nov 6;5:1276. doi: 10.3389/fpsyg.2014.01276 (PMC4222330; doi:10.3389/fpsyg.2014.01276)
Supplement: Supplementary file 1 [file Data_Sheet_1.DOCX]

***Supplementary Material***

**Does cleanliness influence moral judgments? Response effort moderates the effect of cleanliness priming on moral judgments**

**Jason L. Huang^1^***

^1^Department of Psychology, Wayne State University, 5057 Woodward Ave., Detroit, MI, 48202, USA.

*** Correspondence:** Dr. Jason Huang, Department of Psychology, Wayne State University, 5057 Woodward Ave., Detroit, MI, 48202, USA.

jasonhuang@wayne.edu

1. **Pilot Study 1**

Pilot Study 1 involves exploratory analysis on JCD’s Experiment 1. Note that the result below reflects only one of several potential ways to define low response effort subsample, which provided the closest support for the cleanliness hypothesis. This pilot study only served to inform the hypothesis on response effort’s moderating effect, and should not be interpreted as confirmatory evidence in support of the hypothesis.

As a direct replication of SBH’s priming experiment, JCD collected data from 208 undergraduate students (*n*_neutral_ = 102, *n*_cleanliness_ = 106) from Michigan State University. In individual sessions, participants completed a 40-item sentence unscrambling task before evaluating six moral judgment vignettes. The two priming conditions differed only on the sentence unscrambling tasks (neutral words versus cleanliness-related words). Ratings on the six vignettes (i.e., “dog”, “trolley”, “wallet”, “plane”, “résumé”, and “kitten”) and the composite of the six ratings represent the focal dependent variables, which were based on a 10-point scale (0 = *perfectly OK*; 9 = *extremely wrong*).

Exploring the number of correct sentences unscrambled as a proxy for response effort, I identified a low response effort subsample (*n* = 57 who unscrambled 38 or fewer correct sentences; 27% of the full sample). Independent samples *t* tests revealed a pattern consistent with SBH (see Table S1). In this subsample of participants who presumably exerted less effort to complete each sentence scrambling item correctly, the cleanliness condition had a less extreme composite moral judgment than the neutral condition, *d* = -0.41. Across the six vignettes, *d*s ranged from -0.01 to -0.60. In particular, the difference reached statistical significance for the “*kitten*” vignette, *d* = -0.60, *p* = .03. Coincidentally, this vignette was the only one in SBH’s Experiment 1 to receive significantly different ratings between conditions. Despite the overall null finding, there was tentative support in the low response effort subsample in JCD’s data for the expected difference between the cleanliness and neutral conditions.

**Supplementary Table 1. Mean ratings for moral vignettes in low response effort subsample (*n* = 57) in Pilot Study 1**

| Condition | Composite rating |  | Individual vignette rating | | | | | |
| --- | --- | --- | --- | --- | --- | --- | --- | --- |
|  |  |  | Dog | Trolley | Wallet | Plane | Résumé | Kitten |
| **Identified low response effort subsample, *n*_cleanliness_ = 34, *n*_neutral_ = 23** | | | | | | | | |
| Cleanliness | 6.19 (1.31) |  | 7.29 (2.56) | 3.15 (2.38) | 6.50 (2.06) | 6.56 (2.78) | 6.59 (1.91) | 7.03 (2.58) |
| Neutral | 6.64 (0.74) |  | 7.52 (1.88) | 3.17 (2.15) | 6.91 (2.37) | 6.87 (2.40) | 7.17 (2.35) | 8.22 (1.44) |
| *t* | -1.52 |  | -0.36 | -0.04 | -0.70 | -0.44 | -1.04 | -2.22^*^ |
| *p* | 0.13 |  | 0.72 | 0.97 | 0.49 | 0.66 | 0.31 | 0.03 |
| *D* | -0.41 |  | -0.10 | -0.01 | -0.19 | -0.12 | -0.28 | -0.60 |
| *d*_LL_ | -0.94 |  | -0.63 | -0.49 | -0.72 | -0.65 | -0.81 | -1.14 |
| *d*_UL_ | 0.13 |  | 0.43 | 0.48 | 0.34 | 0.41 | 0.25 | -0.06 |

*Note*. ^*^ *p* < .05. Standard deviations are presented in parentheses.

*d*_LL_ and *d*_UL_: lower and upper limits of the 95% CI for *d,* computed using syntax provided by Wuensch (2012).

1. **Pilot Study 2**

Pilot Study 2 involves exploratory analysis on another replication of SBH’s Experiment 1. Similar to Pilot Study 1, the result below represents but one way to define low response effort subsample that yielded close support for the cleanliness hypothesis. Again, this pilot study provided input for the development of the hypothesized moderating effect for response effort, and does not offer any confirmatory evidence.

Lee (2014) reported an online replication of SBH’s Experiment 1 using data from 90 individuals (*n*_cleanliness_ = 42, *n*_neutral_ = 48) recruited from Mechanical Turk (Mturk). The replication design followed SBH but differed on two aspects. First, unlike individual sessions in a laboratory, Lee’s study was conducted online, and thus did not have the same controlled environment across participants. Second, instead of underlining three of four words to form a sentence, participants typed in the sentences directly.

I calculated *survey duration* (rounded to the minute due to the time format in Lee’s (2014) data) as a proxy of participants’ response effort. A short duration likely indicates that the respondent completed the study with limited effort. Independent samples *t* tests in the low response effort subsample (Duration ≤ 8 minutes; *n* = 28; 31% of the full sample) resulted in a pattern of results consistent with SBH, with moderate difference between the conditions on the composite moral judgment, *d* = -0.69, *p* = .08. On the six vignettes, *d* ranged from 0.03 to -0.91 (see Table S2). In particular, the difference on the “*dog*” scenario reached statistical significance, *d* = -0.91, *p* = .02. Similar to Study 1, tentative support for the cleanliness hypothesis was identified in the low response effort subsample.

**Supplementary Table 2. Mean ratings for moral vignettes in low response effort subsample (*n* = 28) in Pilot Study 2**

| Condition | Composite rating |  | Individual vignette rating | | | | | |
| --- | --- | --- | --- | --- | --- | --- | --- | --- |
|  |  |  | Dog | Trolley | Wallet | Plane | Résumé | Kitten |
| **Identified low response effort subsample, *n*_cleanliness_ = 14, *n*_neutral_ = 14** | | | | | | | | |
| Cleanliness | 4.64 (1.56) |  | 3.43 (2.53) | 3.21 (2.52) | 5.21 (2.22) | 5.86 (1.99) | 4.64 (2.06) | 5.50 (3.11) |
| Neutral | 5.65 (1.37) |  | 5.93 (2.92) | 3.79 (2.33) | 5.79 (2.72) | 5.79 (2.33) | 5.57 (2.14) | 7.07 (2.50) |
| *t* | -1.83^†^ |  | -2.42^*^ | -0.62 | -0.61 | 0.09 | -1.17 | -1.48 |
| *p* | 0.08 |  | 0.02 | 0.54 | 0.55 | 0.93 | 0.25 | 0.15 |
| *D* | -0.69 |  | -0.91 | -0.24 | -0.23 | 0.03 | -0.44 | -0.56 |
| *d*_LL_ | -1.45 |  | -1.69 | -0.98 | -0.97 | -0.71 | -1.19 | -1.31 |
| *d*_UL_ | 0.08 |  | -0.13 | 0.51 | 0.52 | 0.77 | 0.31 | 0.20 |

*Note*. ^*^ *p* < .05; ^†^ *p* < .10. Standard deviations are presented in parentheses.

*d*_LL_ and *d*_UL_: lower and upper limits of the 95% CI for *d,* computed using syntax provided by Wuensch (2012).

1. **Supplementary Analysis in Study 1**

In Study 1, a more stringent exclusion of IER cases was done by screening respondents who (a) worked faster than 675 wpm on any of the six moral judgment vignettes, (b) answered incorrectly to either of the two quality screening items from Johnson et al. (2014), and (c) responded incorrectly to more than two of the five IER items adopted from Huang et al. (2014). A final sample of 189 respondents (*n*_cleanliness_ = 96, *n*_neutral_ = 93) was retained after applying this set of exclusion criteria. The low response effort subsample consisted of 95 respondents (*n*_cleanliness_ = 43, *n*_neutral_ = 52) based on a median split on survey duration. In this low response effort subsample, the priming condition had a lower moral composite rating than the neutral condition, *t*(93) *=* -2.00, *p =* .048, *d* = -0.41 (see Table S3).

**Supplementary Table 3. Mean ratings for moral vignettes in Study 1 with a stringent screening of IER (*N* = 189)**

| Condition | Composite rating |  | Individual vignette rating | | | | | |
| --- | --- | --- | --- | --- | --- | --- | --- | --- |
|  |  |  | Dog | Trolley | Wallet | Plane | Résumé | Kitten |
| **Full sample, *n*_cleanliness_ = 96, *n*_neutral_ = 93** | | | | | | | | |
| Cleanliness | 5.94 (1.54) |  | 6.39 (2.81) | 2.61 (2.24) | 6.65 (2.68) | 7.00 (2.63) | 5.85 (2.69) | 7.13 (2.50) |
| Neutral | 6.24 (1.40) |  | 6.69 (2.84) | 3.27 (2.66) | 6.45 (2.52) | 7.13 (2.46) | 6.55 (2.40) | 7.37 (2.33) |
| *t* | -1.42 |  | -0.74 | -1.83^†^ | 0.51 | -0.35 | -1.87^†^ | -0.68 |
| *p* | 0.16 |  | 0.46 | 0.07 | 0.61 | 0.73 | 0.06 | 0.50 |
| *D* | -0.21 |  | -0.11 | -0.27 | 0.07 | -0.05 | -0.27 | -0.10 |
| *d*_LL_ | -0.49 |  | -0.39 | -0.55 | -0.21 | -0.34 | -0.56 | -0.38 |
| *d*_UL_ | 0.08 |  | 0.18 | 0.02 | 0.36 | 0.23 | 0.01 | 0.19 |
| **Identified low response effort subsample, *n*_cleanliness_ = 52, *n*_neutral_ = 43** | | | | | | | | |
| Cleanliness | 5.50 (1.63) |  | 5.98 (2.83) | 2.51 (1.88) | 5.79 (3.01) | 6.28 (2.67) | 5.51 (2.77) | 6.93 (2.57) |
| Neutral | 6.12 (1.38) |  | 6.48 (2.88) | 3.15 (2.64) | 6.65 (2.43) | 6.60 (2.45) | 6.48 (2.50) | 7.35 (2.49) |
| *t* | -2.00^*^ |  | -0.86 | -1.38 | -1.55 | -0.60 | -1.79^†^ | -0.80 |
| *p* | 0.05 |  | 0.40 | 0.18 | 0.13 | 0.55 | 0.08 | 0.43 |
| *D* | -0.41 |  | -0.18 | -0.28 | -0.32 | -0.12 | -0.37 | -0.16 |
| *d*_LL_ | -0.82 |  | -0.58 | -0.69 | -0.72 | -0.53 | -0.78 | -0.57 |
| *d*_UL_ | <0.00 |  | 0.23 | 0.12 | 0.09 | 0.28 | 0.04 | 0.24 |

*Note*. ^*^ *p* < .05; ^†^ *p* < .10. Standard deviations are presented in parentheses.

*d*_LL_ and *d*_UL_: lower and upper limits of the 95% CI for *d,* computed using syntax provided by Wuensch (2012).

1. **Supplementary Analysis in Study 2**

A more stringent exclusion of IER cases was also performed in Study 2 (see criteria above), resulting in a final sample of 374 individuals (*n*_cleanliness_ = 177, *n*_neutral_ = 197). A 2 (neutral vs. cleanliness) × 2 (low vs. high response effort, *n*s = 183 and 191, respectively) factorial ANOVA revealed two nonsignificant main effects (*F*_priming_ = 0.13, *η_p_*^2^ = .00; *F*_effort_ = 1.69, *η_p_*^2^ = .01; MSE = 2.34) and a significant interaction (*F* = 4.24, *p* = .04, *η_p_*^2^ = .01). In the low response effort condition, the simple effect of cleanliness priming on composite moral ratings was negative as expected, *d* = -.27, whereas in the high response effort condition, cleanliness priming had a weak yet nonsignificant positive simple effect, *d* = 0.17 (see Table S4). In general, the findings based on the more stringent screening mirrored those reported in the paper.

**Supplementary Table 4. Mean ratings for moral vignettes in Study 2 with a stringent screening of IER (*N* = 374)**

| Condition | Composite rating |  | Individual vignette rating | | | | | |
| --- | --- | --- | --- | --- | --- | --- | --- | --- |
|  |  |  | Dog | Trolley | Wallet | Plane | Résumé | Kitten |
| **Full sample, *n*_cleanliness_ = 177, *n*_neutral_ = 197** | | | | | | | | |
| Cleanliness | 6.19 (1.53) |  | 6.45 (2.86) | 2.79 (2.14) | 7.02 (2.45) | 7.30 (2.34) | 6.18 (2.64) | 7.41 (2.50) |
| Neutral | 6.25 (1.55) |  | 7.03 (2.66) | 2.99 (2.70) | 6.69 (2.75) | 7.17 (2.47) | 6.12 (2.76) | 7.53 (2.30) |
| *t* | -0.39 |  | -2.03^*^ | -0.79 | 1.25 | 0.53 | 0.21 | -0.51 |
| *p* | 0.69 |  | 0.04 | 0.43 | 0.21 | 0.60 | 0.84 | 0.61 |
| *D* | -0.04 |  | -0.21 | -0.08 | 0.13 | 0.05 | 0.02 | -0.05 |
| *d*_LL_ | -0.24 |  | -0.41 | -0.29 | -0.07 | -0.15 | -0.18 | -0.26 |
| *d*_UL_ | 0.16 |  | -0.01 | 0.12 | 0.33 | 0.26 | 0.22 | 0.15 |
| **Instructed low response effort condition, *n*_cleanliness_ = 80, *n*_neutral_ = 103** | | | | | | | | |
| Cleanliness | 6.13 (1.47) |  | 6.75 (2.70) | 2.70 (1.98) | 6.83 (2.45) | 7.16 (2.30) | 6.03 (2.55) | 7.29 (2.69) |
| Neutral | 6.51 (1.40) |  | 7.54 (2.24) | 2.94 (2.74) | 7.11 (2.63) | 7.23 (2.56) | 6.43 (2.67) | 7.80 (2.04) |
| *t* | -1.80^†^ |  | -2.13^*^ | -0.69 | -0.74 | -0.19 | -1.03 | -1.41 |
| *p* | 0.07 |  | 0.03 | 0.49 | 0.46 | 0.85 | 0.30 | 0.16 |
| *D* | -0.27 |  | -0.32 | -0.10 | -0.11 | -0.03 | -0.15 | -0.21 |
| *d*_LL_ | -0.56 |  | -0.61 | -0.40 | -0.40 | -0.32 | -0.45 | -0.50 |
| *d*_UL_ | 0.03 |  | -0.02 | 0.19 | 0.18 | 0.26 | 0.14 | 0.08 |
| **Instructed high response effort condition, *n*_cleanliness_ = 97, *n*_neutral_ = 94** | | | | | | | | |
| Cleanliness | 6.25 (1.57) |  | 6.21 (2.98) | 2.87 (2.28) | 7.19 (2.45) | 7.41 (2.37) | 6.30 (2.73) | 7.51 (2.35) |
| Neutral | 5.98 (1.66) |  | 6.47 (2.96) | 3.04 (2.66) | 6.22 (2.83) | 7.10 (2.37) | 5.78 (2.83) | 7.24 (2.54) |
| *t* | 1.16 |  | -0.61 | -0.49 | 2.52^*^ | 0.92 | 1.30 | 0.74 |
| *p* | 0.25 |  | 0.54 | 0.62 | 0.01 | 0.36 | 0.20 | 0.46 |
| *D* | 0.17 |  | -0.09 | -0.07 | 0.36 | 0.13 | 0.19 | 0.11 |
| *d*_LL_ | -0.12 |  | -0.37 | -0.36 | 0.08 | -0.15 | -0.10 | -0.18 |
| *d*_UL_ | 0.45 |  | 0.20 | 0.21 | 0.65 | 0.42 | 0.47 | 0.39 |

*Note*. ^*^ *p* < .05; ^†^ *p* < .10. . Standard deviations are presented in parentheses.

*d*_LL_ and *d*_UL_: lower and upper limits of the 95% CI for *d*, computed using syntax provided by Wuensch (2012).

1. **Study 2a: Replication of Study 2**

Study 2a was a direct replication of Study 2. The sample consisted of 529 English-speaking MTurk workers residing in the United States (after removing 75 responses from repeat participants based on worker ID and IP address). Following the same exclusion criteria, 13 respondents were removed due to guessing that cleanliness was central to the study hypothesis, and another 80 respondents were excluded due to excessively fast moral judgment. The remaining 436 (*n*_cleanliness_ = 211, *n*_neutral_ = 225; mean age = 37 years; 68% female; 77% white) were retained in the analysis. The moral judgment composite had an internal consistency reliability of .62.

An initial independent samples *t* test on the composite moral ratings revealed nonsignificant difference across priming conditions, *t*(434) = -0.42, *p* = .38, *d* = -0.08. Further investigation of standardized difference across priming conditions on individual vignettes ranged from -0.15 to 0.09 (see Table S5), with nonsignificant difference on any vignette.

**Table S5. Mean ratings for moral vignettes in study 2a.**

| Condition | Composite rating |  | Individual vignette rating | | | | | |
| --- | --- | --- | --- | --- | --- | --- | --- | --- |
|  |  |  | Dog | Trolley | Wallet | Plane | Résumé | Kitten |
| **Full sample, *n*_cleanliness_ = 211, *n*_neutral_ = 225** | | | | | | | | |
| Cleanliness | 6.10 (1.51) |  | 6.76 (2.96) | 2.94 (2.60) | 6.45 (2.68) | 7.09 (2.44) | 6.06 (2.45) | 7.30 (2.33) |
| Neutral | 6.23 (1.50) |  | 6.86 (2.70) | 2.73 (2.38) | 6.76 (2.71) | 7.16 (2.55) | 6.43 (2.60) | 7.42 (2.33) |
| *t* | -0.87 |  | -0.37 | 0.90 | -1.22 | -0.27 | -1.53 | -0.53 |
| *p* | 0.38 |  | 0.71 | 0.37 | 0.22 | 0.79 | 0.13 | 0.60 |
| *D* | -0.08 |  | -0.04 | 0.09 | -0.12 | -0.03 | -0.15 | -0.05 |
| *d*_LL_ | -0.27 |  | -0.22 | -0.10 | -0.30 | -0.21 | -0.33 | -0.24 |
| *d*_UL_ | 0.10 |  | 0.15 | 0.27 | 0.07 | 0.16 | 0.04 | 0.14 |
| **Instructed low response effort condition, *n*_cleanliness_ = 111, *n*_neutral_ = 111** | | | | | | | | |
| Cleanliness | 5.88 (1.37) |  | 6.69 (3.00) | 2.58 (2.41) | 6.18 (2.70) | 6.90 (2.52) | 5.77 (2.42) | 7.15 (2.35) |
| Neutral | 6.39 (1.35) |  | 7.06 (2.61) | 3.01 (2.59) | 7.15 (2.55) | 7.14 (2.45) | 6.29 (2.67) | 7.68 (2.11) |
| *t* | -2.78^**^ |  | -0.98 | -1.29 | -2.76^**^ | -0.70 | -1.50 | -1.77 |
| *p* | 0.01 |  | 0.33 | 0.20 | 0.01 | 0.48 | 0.14 | 0.08 |
| *D* | -0.37 |  | -0.13 | -0.17 | -0.37 | -0.09 | -0.20 | -0.24 |
| *d*_LL_ | -0.64 |  | -0.39 | -0.44 | -0.64 | -0.36 | -0.46 | -0.50 |
| *d*_UL_ | -0.11 |  | 0.13 | 0.09 | -0.10 | 0.17 | 0.06 | 0.03 |
| **Instructed high response effort condition, *n*_cleanliness_ = 100, *n*_neutral_ = 114** | | | | | | | | |
| Cleanliness | 6.35 (1.62) |  | 6.84 (2.92) | 3.35 (2.75) | 6.74 (2.64) | 7.31 (2.35) | 6.37 (2.46) | 7.47 (2.32) |
| Neutral | 6.07 (1.62) |  | 6.67 (2.79) | 2.46 (2.12) | 6.38 (2.81) | 7.18 (2.66) | 6.56 (2.52) | 7.17 (2.51) |
| *t* | 1.25 |  | 0.44 | 2.64^**^ | 0.97 | 0.36 | -0.56 | 0.91 |
| *p* | 0.21 |  | 0.66 | 0.01 | 0.33 | 0.72 | 0.58 | 0.36 |
| *D* | 0.17 |  | 0.06 | 0.36 | 0.13 | 0.05 | -0.08 | 0.13 |
| *d*_LL_ | -0.10 |  | -0.21 | 0.09 | -0.14 | -0.22 | -0.35 | -0.14 |
| *d*_UL_ | 0.44 |  | 0.33 | 0.63 | 0.40 | 0.32 | 0.19 | 0.39 |

*Note*. ^**^ *p* < .01. Standard deviations are presented in parentheses.

*d*_LL_ and *d*_UL_: lower and upper limits of the 95% CI for *d*, computed using syntax provided by Wuensch (2012).

A 2 (neutral vs. cleanliness) × 2 (low vs. high response effort) factorial ANOVA was conducted to evaluate response effort’s moderating role. The ANOVA revealed two nonsignificant main effects (*F*_priming_ = 0.65, *η_p_*^2^ = .00; *F*_effort_ = 0.26, *η_p_*^2^ = .00; MSE = 2.24) and a significant interaction (*F* = 7.53, *η_p_*^2^ = .02). Follow-up simple effects supported the hypothesis: The cleanliness condition had a lower mean composite rating than the neutral condition, *d* = -0.37, *p* = .006 in the low response effort condition, whereas the trend was reversed but nonsignificant in high response effort condition, *d* = 0.17, *p* = .21 (see Table S5).

Similar to Study 2, the response effort manipulation did not result in significant difference on survey duration, *t*(434) = -0.13, *d* = -0.01, *p* = .89.
